# Attention Bias and Recognition of Sexual Images in Depression

**DOI:** 10.3390/ijerph18168880

**Published:** 2021-08-23

**Authors:** Ondřej Novák, Klára Bártová, Kateřina Klapilová

**Affiliations:** 1 Faculty of Humanities, Charles University, 182 00 Prague, Czech Republic; klara.bartova@fhs.cuni.cz (K.B.); Katerina.Klapilova@nudz.cz (K.K.); 2 Laboratory of Evolutionary Sexology and Psychopathology, National Institute of Mental Health, 250 67 Klecany, Czech Republic

**Keywords:** attention bias, sexual response, sexuality, depression, dot probe task, recognition

## Abstract

Depression greatly affects sexuality. Theoretical and empirical evidence account for the existence of attention bias to sex-related stimuli. This attention bias might be impaired in depression, resulting in sexual problems. A sample of 13 patients with depression and 13 matched healthy controls were tested using the dot-probe and picture recognition task to measure attention to erotic images. No difference in attention to sex-related stimuli (*ω*^2^ = 0, *p* = 0.22) and in memory bias (*ω*^2^ = 0, *p* = 0.72) was found between the two groups. Explorative analyses were conducted to identify the sexual content-induced delay effect in the data, assess variability differences, and compare trial-level bias score-based indexes between groups. Across all analyses, there was little evidence for depression affecting sexual-related cognitive processing, and even this might be explained by other means. Our results suggest that restrained attention is probably not the main factor behind sexual problems in depression.

## 1. Introduction

Clinically significant depression is closely associated with severe changes in sexuality. According to recent meta-analysis, males and females with depression are 1.7 times more likely to suffer from sexual dysfunction, such as lack or loss of sexual desire, lack of sexual pleasure, orgasmic dysfunction, premature ejaculation, vaginism, dyspareunia, or failure of genital response [1]. Erectile dysfunction alone affects males with depression 1.4 times more that those without depression [2]. Even subclinical levels of depressive symptoms negatively affect sexuality in both females [3] and males [4,5]. While the strength of association between depression and sexuality is well explored, its exact mechanisms are poorly understood.

Cognitive theories see depression as a result of maladaptive cognitions [6]. It is a two-way interaction: mood-congruent information processing biases shape attention, memory and interpretations and these cognitions conversely fuel depressive feelings [7,8]. Evolutionary approaches claim that depression is rooted in the downregulation of positive affect systems. In this view, depression is maladaptive expression of defensive strategies that evolved in ancestral human environments. Its purpose revolves around conservation of energy and self-protection by not pursuing goals which are out of control or which are a potential source of severe social conflict—one of such goals being sexual reproduction [9]. Both perspectives highlight the role of attention in steering the experience away from positive and toward negative incentives, thoughts, and interpretations.

Indeed, patients with depression suffer from several cognitive shortcomings. They tend to be slower in psychomotor speed tasks, have generally worse attention, cognitive flexibility, visual learning, and memory [10]. Depressive symptoms also impair executive functions such as working memory updating, mental set shifting and response inhibition [11,12]. Besides these cognitive impairments, depressive patients attend to emotionally loaded stimuli differently than healthy controls. In a meta-analysis of studies using dot-probe and emotional Stroop task paradigms, Perkham et al. [8] presented evidence for a relatively strong attention bias toward negative stimuli (d = 0.37) in depression. Although patients with depression exhibited less attention bias toward positive stimuli than healthy controls (d = −0.23), most of the positive stimuli used contained smiling faces (e.g., [13]), positive-tuned words (e.g., [14]) and pictures of puppies and flowers (e.g., [15]). While nothing is inherently wrong with such stimuli, they are rather weak in terms of both valence and arousal as compared to romantic and sex-related stimuli [16,17,18]. If depression causes attention to avoid general positive stimuli, such an effect should only be pronounced with more potent stimuli.

From an evolutionary point of view, sexual stimuli are highly salient and are assumed to be processed with high priority [19,20]. The effect of sex-related stimuli on human attention in healthy participants is indeed well documented: humans pay more attention to sex-related advertisements [21], to preferred romantic partners (e.g., heterosexual males [22,23], males with a pedophilic preference [24], bisexual males and females [25]) and to erotic pictures (e.g., Doornwaard et al., Kagerer et al., and Mechelmans et al. [26,27,28]; for a review see Novák et al. [29]). Recent meta-analysis assessed that the overall attention bias to, and distractibility by, sexual cues are of medium effect (*g_z_* = 0.49 [20]). Additionally, the SCID (sexual content-induced delay) effect of a general slowing in reaction times has been observed across various studies employing sex-related stimuli [30,31]. Since sexual arousal has been previously conceptualized as an emotional state [32], sex-related cues present strong appetitive stimuli and attention to them might be affected by depression in similar ways as to other positive stimuli—in a reduction in positivity bias.

Nearly all theoretical concepts which operate with attention and sexuality assume some form of attention bias. According to models of sexual dysfunction, attention plays a crucial role in facilitating or inhibiting sexual arousal. Orienting attention toward sex-related stimuli and sustaining attention is required in eliciting physiological and subjective sexual arousal, whereas directing attention to non-sexual cognitions inhibits the arousal or prevents it from appearing. Such influence is mostly carried out by unconscious biases, although it is possible to control this voluntarily [33,34]. The dual control model of sexual reaction presents evidence that an individual’s propensity to focus attention toward or away from sex-related stimuli might explain a large part of interindividual variability in sexual excitability [35]. Both these models suggest that individuals with sexual dysfunction might direct attention away from sexual cues and toward non-sexual and anxiety inducing thoughts (mostly performance-related in the general population [33]). Depression might work in a similar way in sexual functioning, directing attention toward depressive cognitions even in the presence of salient sex-related stimulus.

In the present study we compare the cognitive biases to sex-related stimuli in patients with depression and healthy controls. The dot-probe task (DPT) paradigm is used to assess attention bias. DPT is a gold standard in the field [9,20] and allows the distinction between the SCID effect and visuospatial attention [31]. Together with attention bias, memory bias will be measured using simple picture recognition task (PRT) as a secondary and indirect way to measure attention bias. Successful recognitions are a good measure for how much attention was paid to which stimulus category by participants. Moreover, they are independent from DPT results [29]. Following the literature review, we propose that patients with depression will have significantly lower attention bias and memory bias to sex-related stimuli than healthy participants. Previously, Prause et al. [36] found a negative relationship between attention bias and the sexual excitation scale [37,38] in healthy participants (*r* = −0.44), showing that the greater the excitation scale score, the slower were participants in detecting the probe when it replaced a sexual picture. We seek to replicate their findings in both healthy controls and patients with depression. Moreover, we propose that patients with depression will score lower on the sexual excitation scale and higher on both sexual inhibition scales. Lastly, we hypothesize that there will be negative relationship between attention bias and both sexual inhibition scales for all participants.

## 2. Materials and Methods

### 2.1. Participants

Patients suffering from various forms of depression (F32.0 to F34.1, according to ICD-10 [39]), were recruited for the study. Inclusion criteria included heterosexual males and females between ages 18 and 70, with ongoing depressive episodes, within two weeks from the initiation of medical treatment with antidepressants (median of 59% of weighted mean dose of 40 mg fluoxetine; computed using [40]) to minimize the effect, as antidepressants greatly affect sexuality in patients [41]). Exclusion criteria included any comorbid disease which might affect emotional experience, cognitive or psychomotor performance (such as dementia, cognitive syndromes, or psychotic symptoms). All participants were offered a monetary compensation of 200 CZK (app. 7.6 EUR) for participation in the study. The study ran from 2018 to 2020. Out of 88 eligible contacted patients only 14 accepted the invitation to be part of the study. Later, one patient was excluded from the study (see Data Analysis) resulting in 13 patients in the experimental group (7 females, *M_age_* = 48.38, *SD_age_* = 16.13). The same number of healthy controls closely matched in terms of sex, age, education, and relationship status (7 females, *M_age_* = 45.77, *SD_age_* = 16.20) was recruited via social sites and a volunteer list. All participants stated, via the Kinsey scale, that they were heterosexual (0 or 1 on Kinsey scale). Patients with depression had a greater score in the Screening Questionnaire for Psychiatric Disorders, BDI–II [42] (*M* = 21.92, *SD* = 11.59) than healthy controls (*M* = 6.31, *SD* = 3.47; *t*(14.14) = 4.66, *p* < 0.001). BDI-II scores of healthy controls were comparable to those of the general healthy Czech population [43] (*Z* = −1.38, *p* = 0.17).

### 2.2. Measures

All methods in their presented forms (except for Sexual Excitation and Sexual Inhibition scales; SIS/SES [37,38,44]) were used in our previous study, focused on a healthy population [29]. Both DPT and PRT were run using E-Prime software package 2.0 [45], and all questionnaires were in paper form.

#### 2.2.1. Dot-Probe Task

Each trial of the DPT started with a 500, 750, 1000 or 1500 milliseconds long intertrial black screen, which was followed by a fixation cross for a duration of 1000 ms. On the next screen, laterally randomized sexual and neutral pictures were presented on both sides of the screen for 500 ms. Both pictures disappeared and one of them was replaced by a probe in the form of an arrowhead (pointing left or right). The probe stayed on the screen until the participant responded. Participants were instructed to quickly press the key assigned to where the probe was pointing (Q for left, P for right). Position of the probe, its direction, and which picture category was replaced were all randomized. The DPT started with 20 training trials and continued with two blocks consisting of 50 experimental trials. Each block contained the identical but randomly paired stimuli.

#### 2.2.2. Picture Recognition Task

A yes/no task paradigm was used for the PRT. Participants were presented with a series of pictures. Signal trials contained pictures previously encountered in the DPT, and noise trials contained new pictures with similar content to those in signal trials (see Materials). Noise trials made up 62% of presented stimuli. Participants were instructed to indicate whether they previously saw the pictures by pressing assigned keys (P for “seen”, Q for “not seen”). The picture remained on the screen until a response was recorded and participants were encouraged to go through the PRT at their own pace.

#### 2.2.3. Questionnaires

All participants completed a short battery of questionnaires including the Screening Questionnaire for Psychiatric Disorders. The Beck depression inventory for adults (BDI–II [42]) is a reliable tool for determining the intensity of depression. It consists of 21 items covering various depressive symptoms such as changes in sleep or loss of interest. Sexual Inhibition/Sexual Excitation Scales [37,38,44] are based on the dual control model of sexual reaction and measures three higher-order factors: sexual excitation (SES), sexual inhibition due to performance failure (SIS1), and sexual inhibition due to performance consequences (SIS2). Back-translated Czech versions of BDI-II [43] and SIS/SES [46] were used. Both questionnaires have high levels of inner consistency in their Czech versions (Cronbach’s alpha of 0.92 for BDI-II and from 0.78 to 0.88 for SIS/SES factors).

### 2.3. Materials

For both DPT and PRT, there were 50 pictures in sexual condition (naked males, females, and heterosexual couples engaging in sexual activities), 50 pictures in neutral condition (seven clothed males for female version, seven clothed females for male version and 43 heterosexual couples for both versions), and 40 training pictures (clothed males). For the PRT, another 255 pictures were used as distractors (the same content as in sexual, neutral, and training conditions). All pictures selected for the study were standardized in their sexual excitation potential for both sexes. The evaluation procedure has been described in detail elsewhere [29].

### 2.4. Procedure

Patients from the ambulance and inpatient ward of the National Institute of Mental Health were contacted by their physician or the researcher and were offered participation in the study. Healthy participants were recruited using social sites and a volunteer list. The study took place in an isolated room. Participants received detailed information about the study, their eventual questions were answered, and they signed the informed consent form. At the beginning, each participant completed the Screening Questionnaire for Psychiatric Disorders, BDI–II, and SIS/SES. Next, each participant was seated in front of a personal computer and the DPT and PRT were administered. Finally, each participant was debriefed and received their monetary compensation before leaving. The entire procedure took approximately 30 min.

### 2.5. Ethics Statement

Participants were informed about the entire procedure and signed an informed consent form. They were advised of the fact that the tasks would feature explicit sexual material. All experimental procedures were in accordance with the Helsinki Declaration and the study was approved by the Ethics Committee of the National Institute of Mental Health, Klecany (No 47/16).

### 2.6. Data Analysis

Data analyses were performed using R [47] and JASP [48]. DPT reaction times (RT) were trimmed by incorrect responses (0.02% of all data) and values 4 SD above and below the group mean (0.005% of all data). Following these criteria, a median number of one trial (ranging 0 to 14) was removed from each participant. There was no difference in error rates in DPT between the experimental and control groups (*t*(24) = 1.24, *p* = 0.23). Moreover, only participants with error rates lower than 30% were included in the analysis, which resulted in exclusion of one female patient with depression. Descriptive statistics of mean, median and SD for both groups are presented in Table 1.

No normality violations were detected using the Shapiro–Wilk test (*p* > 0.05). Mixed ANOVA was performed on RT with one within-subject factor (Trial condition: sexual and neutral) and one between-subject factor (Group: experimental and control).

Attention bias index (AB index) for the DPT was computed for each participant by subtracting the mean reaction time in sex target trials from the mean reaction time in neutral target trials. Positive values indicate vigilance to sexual pictures, and negative values indicate avoidance of sexual pictures. AB index scores were not significantly different from 0 for experimental (*M* = −18.18, *SD* = 57.66, *p* = 0.28) nor for control group (*M* = 5.70, *SD* = 37.41, *p* = 0.59). The relationship between AB index and summed scores of the questionnaires was tested using Pearson correlation coefficient separately for each group.

The PRT was evaluated using signal detection theory [49]. Hit rates for each picture category (sexual, neutral, training) and false alarm rates were used in the analysis (Table 2). The false alarm rates were low for both experimental (*M* = 18.0%, *SD* = 16.3%) and control (*M* = 15.9%, *SD* = 16.5%)) group and no significant difference was found between them (*p* = 0.64). Mixed ANOVA was performed for hit rates with one within-subject factor (Trial condition: sexual, neutral, training) and one between-subject factor (Group: experimental and control).

Questionnaire scores were computed and compared between experimental and control group using Welch *t*-tests. Descriptive statistics of mean, median and SD of questionnaires scores for both groups are presented in Table 3. Additionally, the potential relationship between BDI-II and SES was explored using the Pearson correlation coefficient as suggested by one of the reviewers.

Due to the rather small sample size, we set the alpha level of statistical significance at 0.1 to increase the sensitivity of the analyses [50] and calculated effect sizes and confidence intervals for all tests.

## 3. Results

### 3.1. Dot-Probe Task

**Figure 1:**
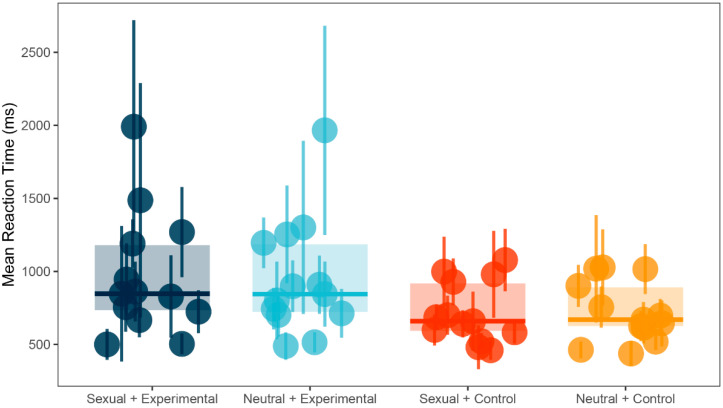
Dot-probe task: mean reaction times (ms) and standard deviations (SD) for both groups and conditions.

There was a significant main effect in between-subject factor Group (*F*(1, 24) = 3.50, *p* = 0.07, *ω*^2^ = 0.05). Post-hoc test using Bonferroni correction showed that control group (*M* = 719.83, *SD* = 207.70) was significantly faster in identifying the probe than the experimental group (*M* = 957.76, *SD* = 409.54; *t*(24) = 1.87, *p* = 0.07, *d* = 0.73, 95% *CI* (−0.07, 1.52)). However, there were no significant differences for within-subject factor Trial condition (*F*(1, 24) = 0.43, *p* = 0.52, *ω*^2^ = 0) or for interaction between the two factors (*F*(1, 24) = 1.57, *p* = 0.22, *ω*^2^ = 0). See Figure 1 for illustration and Table 1 for a summary.

### 3.2. Picture Recognition Task

Mauchly’s test indicated that the assumption of sphericity for the ANOVA was violated in the analysis of hit rates (*χ*^2^(2) = 28.75, *p* < 0.001), which is why degrees of freedom were corrected using Greenhouse–Geisser estimates of sphericity (*ε* = 0.58).

**Figure 2:**
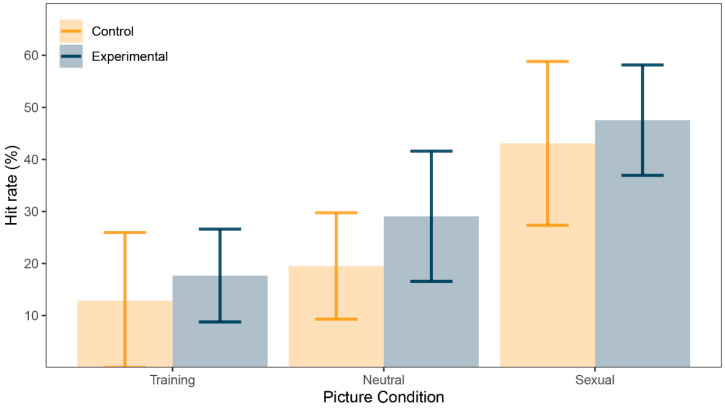
Picture recognition task: correct recognition (%) for training, neutral, and sexual pictures.

There was a significant main effect in within-subject factor Trial condition (*F*(1.17, 28.01) = 20.52, *p* < 0.001, *ω*^2^ = 0.24). Post-hoc tests using Bonferroni correction revealed that sexual pictures’ hit rate (*M* = 45.30%, *SD* = 24.30) was greater than for neutral pictures’ hit rate (*M* = 24.30%, *SD* = 15.30, *d* = 0.88, 95% *CI* (0.42, 1.33), *p* < 0.001), sexual pictures’ hit rate was greater than training pictures’ hit rate (*M* = 15.30%, *SD* = 20.30, *d* = 0.95, 95% *CI* (0.47, 1.40), *p* < 0.001) and neutral pictures’ hit rate was greater than training pictures’ hit rate (*d* = 0.69, 95% *CI* (0.26, 1.11), *p* = 0.002). However, there were no significant differences for between-subject factor Group (*F*(1, 24) = 0.88, *p* = 0.36, *ω*^2^ = 0) or for interaction between the two factors (*F*(1.17, 28.01) = 0.17, *p* = 0.72, *ω*^2^ = 0). See Figure 2 for illustration and Table 2 for summary.

### 3.3. SIS/SES and Associations with AB Index

The only significant differences between experimental and control group were in sexual excitation score (*t*(23.949) = 2.93, *p* = 0.01, *d* = 1.15, 95% *CI* (0.31, 1.98)). Experimental group reported higher levels of sexual excitation (*M* = 51.39, *SD* = 6.01) than control group (*M* = 44.31, *SD* = 6.29). There were no significant differences between the groups in threat of performance failure score (SIS1, *d* = −0.02, *p* = 0.95) or threat of performance consequences score (SIS2, *d* = −0.55, *p* = 0.18). See Table 3 for summary.

For the experimental group, there was only one marginally significant relationship between AB index and sexual excitation score (SES, *r* = −0.48, *p* < 0.10). There were no significant relationships between AB index and threat of performance failure score (SIS1, *r* = −0.12, *p* = 0.70) or threat of performance consequences score (SIS2, *r* = −0.32, *p* = 0.29).

For the control group, there was only one significant relationship between AB index and sexual excitation score (SES, *r* = 0.48, *p* < 0.10). There were no significant relationships between AB index and threat of performance failure score (SIS1, *r* = 0, *p* = 1) or threat of performance consequences score (SIS2, *r* = −0.12, *p* = 0.69).

As a follow up analysis [51], the AB index and SES correlations were compared between experimental and control group. The difference was statistically significant (*Z* = −2.327, *p* = 0.01).

For BDI-II and SES we found a small and non-significant positive relationship be-tween BDI-II and SES in the whole sample (*r* = 0.28, *p* = 0.18) and a medium and non-significant positive relationship in the experimental group (*r* = 0.40, *p* = 0.17). No correlation was performed for control group because of low variability of BDI-II scores in that group.

### 3.4. Explorative Analyses

To better understand the data, a set of explorative analyses was performed. Potential SCID effect was assessed. A trial-to-trial performance graph was created to evaluate the learning rate of both groups and the degree of inter-group variability in response times. Finally, trial level bias score (TL-BS) indexes for intra-personal variability in attention bias were computed as proposed by Zvielli et al. [52].

#### 3.4.1. SCID Effect

**Figure 3:**
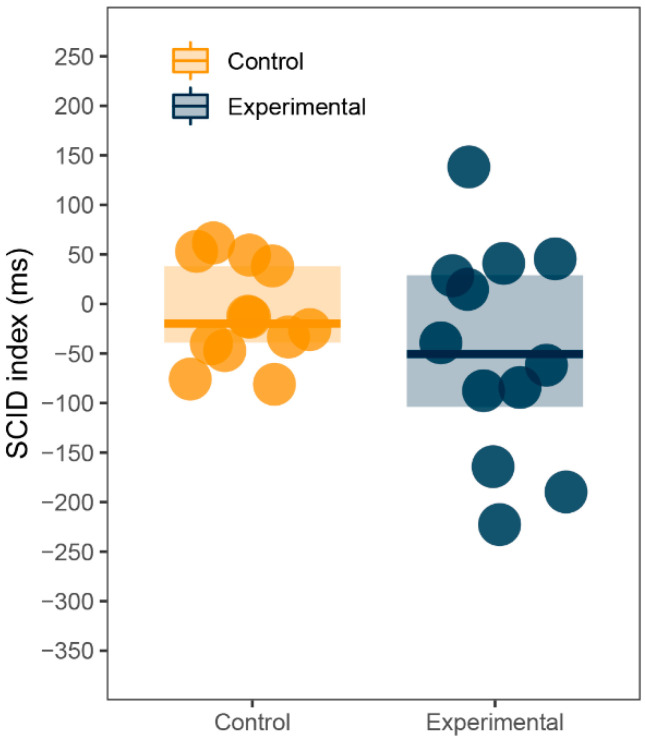
Sexual content induced delay (SCID): SCID index (ms).

Although the study was not designed to assess the SCID effect, the large number of training trials which use only neutral stimuli allows for a comparison between them and sexual trials. If there was a SCID effect in our data, participants would react faster in trials with no sexual content. Reaction time means were computed from the last ten training trials and the first ten sexual trials for each participant. SCID index was calculated by subtracting training trials from sexual trials, as in previous research [53]. Unlike the AB index, positive values indicate slower reactions to sexual pictures and therefore the SCID effect, whereas negative values indicate faster reaction to sexual pictures. Since the index for both groups failed the Shapiro-Wilk normality test (*p* < 0.001), Wilcoxon signed-rank test and Mann-Whitney U test were used. SCID index did not statistically differ from zero for both experimental (*M* = 48.35, *Mdn* = 50.41, *SD* = 108.11, *p* = 0.18) and control (*M* = −18.60, *Mdn* = 13.40, *SD* = 115.61, *p* = 1) group. Additionally, there was no difference in SCID index between groups (*p* = 0.15). See Figure 3 for illustration.

#### 3.4.2. Trial-to-Trial Performance

**Figure 4:**
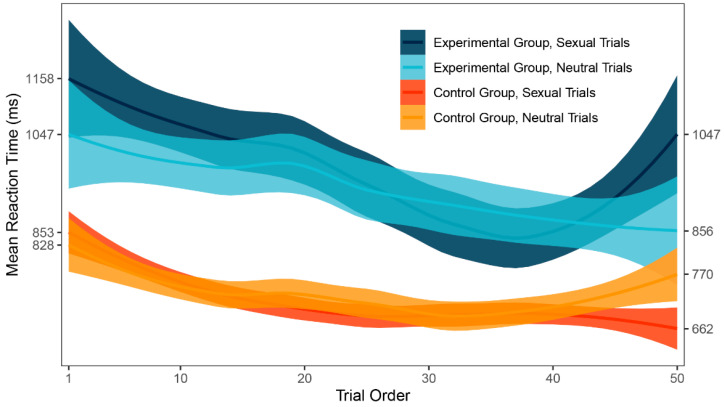
Trial-to-trial performance (ms).

In the main analysis, no significant difference was found between the mean response times to sexual and neutral trials in both experimental and control group. One possible explanation might be that there was an important change in attention bias throughout the task because participants were learning how to ignore sexual stimuli. To evaluate such potential change, local polynomial regression fitting [54] was used on the data to create a smoothed graph (Figure 4), which might then be visually inspected. A learning curve was apparent for both groups in the first part of the task. Additionally, the graph shows a large difference in inter-group variability of response times. There seems to be no attention bias in any part of the DPT for either group.

#### 3.4.3. Trial Level Bias Score Indexes

**Figure 5:**
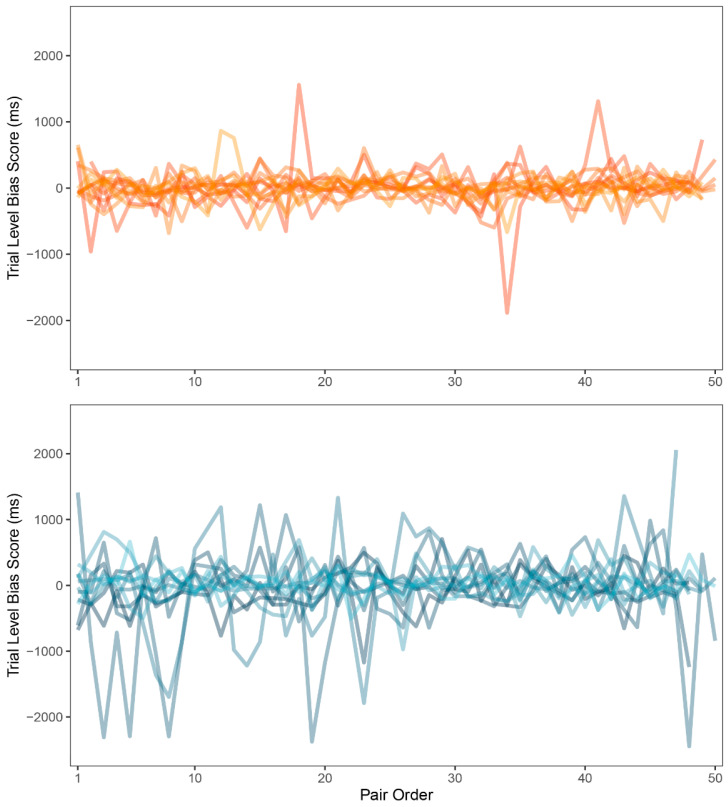
Trial level bias score (TL-BS) variability (ms) for control group (**top**) and experimental group (**bottom**).

Zvielli et al. [52], regarding inconclusive results in attention bias research, suggested that emotional attention or attention bias may not be expressed as a stable or static signal (as measured by attention bias index computed from mean response times). They proposed that attention bias may be expressed in fluctuating, phasic bursts towards or away from the target stimulus category over time. To measure this, they introduced the trial level bias score, a computational methodology based on the common DPT data. TL-BS methodology uses differences in response times of neighboring congruent (i.e., sexual) and incongruent (i.e., neutral) trials instead of the means computed from the whole task duration. While proposed in 2015, this approach was included in general research only recently [55]. The novel methodology includes five indexes of attention bias: mean of positive TL-BS, mean of negative TL-BS, maximum and minimum peak TL-BSs and TL-BS variability index, exploring the temporal variability in the expression of attention bias towards and/or away from the stimuli over the course of the task. All five indexes were computed for each participant (for details of the computation of these indexes see [52]). Because of the normality violations (*p* < 0.05), indexes were compared between groups using the Mann-Whitney U test. Statistically significant differences were found in all but one index. Experimental group (*Mdn* = 228.40) showed larger values in the mean of positive TL-BS than control group (*Mdn* = 140.23; *p* = 0.060; *r_bs_* = −0.44, 95% *CI* (−0.73, −0.03)). Experimental group (*Mdn* = −214.69) also showed smaller values in the mean of negative TL-BS than control group (*Mdn* = −128.05; *p* = 0.03; *r_bs_* = 0.49, 95% *CI* (0.09, 0.76)). Experimental group (*Mdn* = −641.00) had smaller negative peak than control group (*Mdn* = −498.00; *p* = 0.09; *r_bs_* = 0.40, 95% *CI* (−0.03, 0.70)). Experimental group (*Mdn* = 269.77) also showed larger TL-BS variability than control group (*Mdn* = 168.93; *p* = 0.04; *r_bs_* = −0.47, 95% *CI* (−0.74, −0.05)). There was no difference in positive peak values in experimental (*Mdn* = 663.00) and control group (*Mdn* = 603.00; *p* = 0.48; *r_bs_* = −0.17, 95% *CI* (−0.56, 0.27)). See Table 4 for a summary. Finally, variability in TL-BS should be accessible to visual inspection, showing larger changes in reaction times when attention bias is present. For comparison of the TL-BS between-group variability, see Figure 5.

## 4. Discussion

The aim of the current study was to compare the attention and memory biases to sex-related stimuli in patients with depression and healthy controls. Following previous research and theoretical frameworks, we expected the patients to exhibit lower attention and memory biases as measured by DPT and PRT than control group.

While no power analysis was computed before the data collection, sensitivity analysis computed post-hoc for the main hypothesis (*β* = 0.8, *α* = 0.1) showed that the lowest possible difference between the two groups to be reliably revealed—if such difference existed between the respective populations—was 22.97 ms.

Although patients with depression were generally slower (but not less successful) in both cognitive tasks, our main hypothesis was not supported by the data, showing no significant difference in attention bias or memory bias between the two groups. For patients we observed a mean AB index of −18.18 ms. Such a result is indeed in the range of typical attention bias found in various clinical populations [20] and might suggest avoidance of sexual pictures, which does correspond to what we hypothesized. However, the variation in scores was too high to tell reliably.

Nevertheless, we observed rather substantive difference in hit rates for all picture conditions and across the groups. As explored in previous work [29], memory bias is an indicator of attention being paid to the stimulus category in question, since attention is necessary to create a reliable memory of that stimulus. If there is attention bias for sex-related stimuli, we would expect the participants to mostly look at sexual pictures during DPT. Participants were indeed most successful recognizing sexual pictures, achieving a hit rate of 45%. Since neutral pictures were presented to participants twice (once per block) and training pictures only once, we would expect this to be reflected in the data in larger hit rates for neutral than for training pictures. Indeed, neutral pictures were correctly recognized in about one quarter of the time while training pictures produced a very low hit rate of 15%. It should be noted that our results are very similar to data we collected from young adults where sexual pictures were recognized in 48%, neutral pictures in 19% and training pictures in 15% of the cases [29]. Such results suggest that there is indeed a memory bias and quite possibly attention bias hidden in the data. However, there was no difference in any hit rate between the groups. Together with no difference in AB index between the experimental and control groups, these results suggest that there is no difference in attention paid to sex-related stimuli between patients with depression and healthy individuals.

This notion was further supported by the weak relationship between AB index and any of the questionnaire scores. The only significant results came from sexual excitation scores in both groups. There was a major correlation of *r* = 0.48 in the control group, meaning that the greater the sexual excitation score, the greater the AB index. As mentioned earlier, positive values of AB index show faster reactions toward sexual stimuli, meaning that our healthy participants were faster in detecting the probe after it replaced the sexual picture when their sexual excitation score was higher. This rather intuitive result directly contradicts previous findings of Prause et al. [36], who found a negative relationship between AB index and sexual excitation. However, patients with depression did show a negative relationship between the two variables (*r* = −0.48). In their study with 69 healthy participants, Prause et al. explained that participants with higher sexual desire might have had more experience with sexual stimuli and are therefore habituated to it, reducing attentional capture. The same can hardly be said about patients with depression. A more traditional explanation can be offered for negative attention bias in DPT, suggesting that the greater sexual excitation, the greater the effort to avoid wanted yet unavailable stimuli. These results might tap into the cognitive procedures in depression. It could be that the attention bias steering attention away from sexual pictures is only pronounced in patients with higher levels of sexual drive. It is necessary to say, however, that not all of the results support such a narrative. For example, contrary to our hypothesis, patients with depression had significantly greater levels of sexual excitation than healthy controls (*d* = 1.15). We also hypothesized that patients with depression will score higher on inhibition scales (SIS1 and SIS2) reflecting their fear of performance failure and consequences, but there were no other differences between the two groups. Lastly, we found a positive but non-significant relationship between the severity of depression (BDI-II) and individual propensity to sexual excitation (SES).

An argument could be made that while certainly appetitive, erotic stimuli might not necessarily be perceived as positive, thus mixing avoidance of and vigilance to sexual pictures according to individual attitudes toward sexuality or pornography. This issue might be especially pronounced when considering sex differences. As observed in previous studies [56], males and females do have different perceptions and reaction patterns toward sexual stimuli, and it is not unwise to assume such a difference in attention to sexual pictures. However, from the previous studies on the subject, including our recent research with a large sample of healthy university students [29], only one [57] reported any kind of sex differences in DPT. Moreover, while our dataset is too small to statistically assess gender difference while controlling for the influence of depression, visual inspection of the data did not suggest any meaningful partitioning into separate groups of responses which could be identified by the individual’s sex. If there were any sex differences in automatic attention toward sexual pictures, they are probably not measurable by DPT.

So far, our data produced rather anticlimactic or downright confusing results. None of our hypotheses was supported by the data and some values were the exact opposite of what we expected to see. To further explore the dataset, we employed several additional analyses. We assessed the proposed SCID effect [31] using training trials. As the results and Figure 3 show, there was no SCID present in our data, but keeping in mind that the effect is consistent but rather small across different studies, it might be possible for it to emerge in a larger sample size.

Next, trial-to-trial performance was visualized to evaluate the learning curve and the variability in response times between groups. As expected, a learning curve was clearly visible for both groups. The experimental group was generally slower in identifying the probe, which was also shown by our main results, and demonstrated larger inter-group variability in response times. However, large inter-group variability could be a result of inter-individual differences rather than intra-individual. As a remedy to this and in search for a more modern approach to attention bias, we employed trial level bias score (TL-BS), a computational methodology invented by Zvielli et al. [52] in response to inconsistent results in attention bias literature. Groups were compared in the proposed variables and significant differences were found in all but one index. Patients with depression had more extreme positive attention bias values, negative attention bias values, negative attention bias peak, and showed far larger TL-BS variability. Judging by the results, the performance of patients with depression was highly inconsistent. This is also apparent in Figure 5 showing TL-BS variability: performance of healthy participants tended to be much more consistent and without many phasic bursts in attention bias towards or away from the target stimulus category. According to the authors, the fluctuating nature of attention bias scores in our depressive sample suggests that attention was greatly influenced by the stimuli. There is, of course, a very real possibility that even such intra-individual differences were caused mainly by negative depressive symptoms affecting the whole of the participant’s cognitive performance and would occur even out of the sex-related context. To test for such a hypothesis, more trials with two neutral stimuli would be needed for assessment in participants with depression, in order to compute target-free TL-BS values. While an interesting and beneficial methodological approach to attention bias, TL-BS needs to be firstly assessed without the presence of potential cause for bias.

We tried to compensate for the small sample size by exploring the data with novel methods and visual analyses. We found only subtle indices of the presence of attention bias towards or away from sex-related stimuli. While it might be beneficial to explore the topic further with a much larger sample, our extensive results suggest that attention might not be the main reason behind sexual problems in depression, but only a contributing factor at the most.

## 5. Conclusions

Employing two cognitive tasks and several methods of analysis, we assessed potential cognitive bias to sex-related stimuli in a rather small sample of patients with depression compared with healthy controls. Across all analyses, there was little evidence for existence of such bias. Even results pointing in that direction might be very well explained by different sources of influence, such as general negative cognitive impairment in depression. While depression does affect cognition regarding positive stimuli, it appears not to impact sexual-related processing in such a way that we might consider important.

## Figures and Tables

**Table 1:** Dot-probe task: Mean RT (ms), median, and *SD*.

	Experimental Group (*n* = 13)			Control Group (*n* = 13)		
	Mean	Median	*SD*	Mean	Median	*SD*
All trials	957.761	846.031	409.537	719.826	669.490	207.697
Sex target	967.003	847.300	421.457	716.864	659.412	209.253
Neutral target	948.823	844.708	398.664	722.561	670.038	208.125
AB index ^1^	−18.179	−11.048	57.656	5.697	2.859	37.409

^1^ AB index = attention bias index.

**Table 2:** Picture recognition task: Mean hit rates (%), Median, and *SD*.

	Experimental Group (*n* = 13)			Control Group (*n* = 13)		
	Mean	Median	*SD*	Mean	Median	*SD*
Sexual	47.50	48.00	19.50	43.10	42.00	28.90
Neutral	29.10	24.00	23.00	19.50	12.00	18.80
Training	17.70	10.00	16.40	12.90	2.50	24.10
False alarm	18.00	16.40	0.16	15.90	9.80	0.17

**Table 3:** Questionnaires: Mean, Median, and *SD*.

	Experimental Group (*n* = 13)			Control Group (*n* = 13)		
	Mean	Median	*SD*	Mean	Median	*SD*
BDI-II	21.923	21.000	11.586	6.308	6.000	3.473
SES	51.385	51.000	6.007	44.308	46.000	6.290
SIS1	27.000	27.000	3.512	27.077	27.000	3.278
SIS2	28.615	29.000	3.798	31.385	31.000	6.090

**Table 4:** Trial Level Bias Score: Mean positive, mean negative, peak positive, peak negative and variability: Mean RT (ms), median, and *SD*.

	Experimental Group (*n* = 13)			Control Group (*n* = 13)		
	Mean	Median	*SD*	Mean	Median	*SD*
Mean positive	253.831	228.400	171.726	150.684	140.226	75.935
Mean negative	−285.199	−214.692	258.528	−144.009	−128.053	73.809
Peak positive	743.154	663.000	508.258	630.154	603.000	410.404
Peak negative	−1034.692	−641.000	843.780	−543.615	−498.000	442.525
Variability	356.694	269.766	249.224	205.655	168.932	99.390

## Data Availability

Datasets and R scripts of the study are available at OSF website at https://osf.io/hp5wa/ accessed on 13 August 2021.
